# Disruption or reduced expression of the orotidine-5′-decarboxylase gene *pyrG* increases citric acid production: a new discovery during recyclable genome editing in *Aspergillus niger*

**DOI:** 10.1186/s12934-020-01334-z

**Published:** 2020-03-24

**Authors:** Lihui Zhang, Xiaomei Zheng, Timothy C. Cairns, Zhidan Zhang, Depei Wang, Ping Zheng, Jibin Sun

**Affiliations:** 1 College of Biotechnology, Tianjin University of Science & Technology, Tianjin, 300457 China; 2 Tianjin Institute of Industrial Biotechnology, Chinese Academy of Sciences, Tianjin, 300308 China; 3 Key Laboratory of Systems Microbial Biotechnology, Chinese Academy of Sciences, Tianjin, 300308 China; 4 University of Chinese Academy of Sciences, Beijing, 100049 China

**Keywords:** *Aspergillus niger*, Citric acid, *pyrG*, CRISPR/Cas9 system, Tet-on system

## Abstract

**Background:**

*Aspergillus niger* is a filamentous fungus used for the majority of global citric acid production. Recent developments in genome editing now enable biotechnologists to engineer and optimize *A. niger*. Currently, however, genetic-leads for maximizing citric acid titers in industrial *A. niger* isolates is limited.

**Results:**

In this study, we try to engineer two citric acid *A. niger* production isolates, WT-D and D353, to serve as platform strains for future high-throughput genome engineering. Consequently, we used genome editing to simultaneously disrupt genes encoding the orotidine-5′-decarboxylase (*pyrG*) and non-homologous end-joining component (*kusA*) to enable use of the *pyrG* selection/counter selection system, and to elevate homologous recombination rates, respectively. During routine screening of these *pyrG* mutant strains, we unexpectedly observed a 2.17-fold increase in citric acid production when compared to the progenitor controls, indicating that inhibition of uridine/pyrimidine synthesis may increase citric acid titers. In order to further test this hypothesis, the *pyrG* gene was placed under the control of a tetracycline titratable cassette, which confirmed that reduced expression of this gene elevated citric acid titers in both shake flask and bioreactor fermentation. Subsequently, we conducted intracellular metabolomics analysis, which demonstrated that *pyrG* disruption enhanced the glycolysis flux and significantly improved abundance of citrate and its precursors.

**Conclusions:**

In this study, we deliver two citric acid producing isolates which are amenable to high throughput genetic manipulation due to *pyrG/kusA* deletion. Strikingly, we demonstrate for the first time that *A. niger pyrG* is a promising genetic lead for generating citric acid hyper-producing strains. Our data support the hypothesis that uridine/pyrimidine biosynthetic pathway offer future avenues for strain engineering efforts.
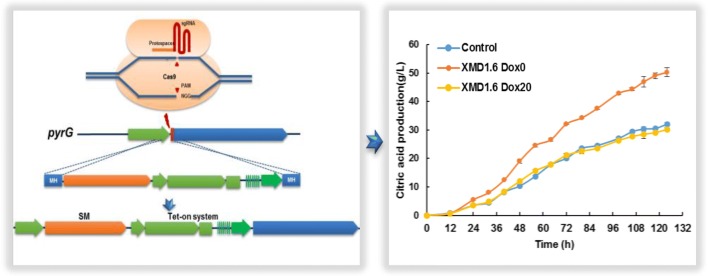

## Background

Citric acid is the most important bulk industrial organic acid with the worldwide market of nearly 2 million tons per year [1] and has been widely applied in various industries [1–3]. *Aspergillus niger* is the important industrial cell factory for the citric acid production, which contributes approximately 80% of world-wide citric acid [4]. Over the past 20 years, numerous technological advances have occurred in the *A. niger* field [5], including the development of recyclable selection markers, disruption of non-homologous end-joining systems for high throughput gene targeting, and, most recently, genome editing [6–13]. Most of these tools and techniques have been developed and optimized in fully sequenced strain backgrounds. This includes isolates CBS 513.88 or ATCC 1015, which are industrial isolates used for glucoamylase and citric acid fermentation respectively [14, 15]. As sequencing and genome editing technologies are increasingly cheap and simple to develop, it is now possible to expand the full *A. niger* toolkit to other industrially harnessed strains. This is an attractive strategy, as optimization efforts in specific isolates can rapidly be applied at a commercial scale. Currently, this toolkit remains a key objective for biotechnologists as genetic leads for generating *A. niger* citric acid hyper-producing isolates are limited [5, 16, 17]. For example, several studies have used gene knock-down of the chitin synthase *chsC* [18] or amino acid transporter *Brsa*-*25* [19] to elevate citric acid titers about 42.6% [18] and 10% [19], respectively. Alternatively, the over-expression of the organic acid transporter *cexA* has been used for hyperproduction [20]. Despite these studies, the majority of genes with potential industrial applications to elevate citric acid production remain hypothetical and lack functional characterization in the laboratory [5, 16, 17].

Arguably, the first and most important technology for genetic manipulation of any given *A. niger* isolate is development of an efficient selection marker for transformation. The orotidine-5′-decarboxylase encoding gene *pyrG* has been widely applied as a recyclable transformation marker in many fungal species [8, 11, 20–23]. The use of this system requires that a strain of interest firstly has *pyrG* disrupted/deleted, generating a uridine auxotrophy as the orotidine-5′-monophosphate (OMP) decarboxylase. PyrG is an essential enzyme involved in uridine biosynthesis [21]. Subsequently, a functional *pyrG* encoded in a transformation cassette serves as an efficient selection marker by restoring prototrophy. Additionally, as PyrG enables catalysis of the non-toxic 5-fluoroorotic acid (5-FOA) to the toxic product as 5-fluorouracil, isolates containing *pyrG* marker can be counter selected. Several *Aspergillus* spp. transformation cassettes are designed to facilitate efficient excision of the *pyrG* selection marker from recipient genomes under 5-FOA counter selection, ultimately generating a fully recyclable marker system. More recently, an additional benefit of using the *pyrG* marker has been demonstrated, as *pyrG* resides in a highly expressed locus on chromosome III [24]. Consequently, the *pyrG* marker and genomic locus are highly convenient for gene over-expression studies.

An additional objective for enabling high throughput gene functional analysis in a strain of interest is disruption/deletion of non-homologous end-joining pathways, most commonly *kusA* in *A. niger* [25]. Deletion of this gene elevates homologous recombination rates, therefore increasing the targeting efficiency of exogenous DNA cassettes with the recipient genome. Recently, concerns regarding genome stability in *kusA* mutants have been disproven by sequencing NHEJ mutants and progenitor controls, confirming their application in biotechnology [26]. In this study, we simultaneously disrupted both *pyrG* and *kusA* by a highly efficient CRISPR/Cas9 system based on 5S rRNA. Surprisingly, during routine screening of the mutant strains and progenitor controls, it demonstrated that the *pyrG* gene is promising target to modulate citric acid production during submerged industrial fermentation.

## Results and discussion

### Gene disruption of *pyrG* and *pyrG/kusA* constructed by CRISPR/Cas9 system in *A. niger*

In yeast and filamentous fungi, the *pyrG* gene has been widely used as nutritional/auxotrophic marker for fungal genetic manipulation [21, 22, 27–30]. With the advantage of counter selection using 5-FOA, *pyrG* could is utilized as the bidirectional selection marker for recyclable genome editing.

**Fig. 1:**
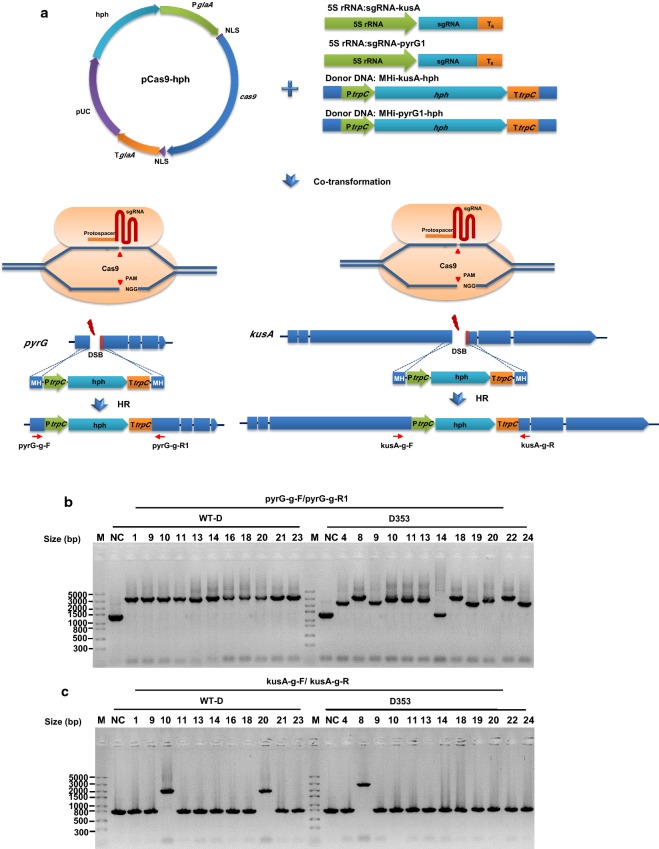
Simultaneous inactivation of *pyrG* and *kusA* in *A. niger* D and D353. **a** Schematic diagram of simultaneous disrupted mutagenesis of both *pyrG* and *kusA* mediated by integrating the donor DNA with 40-bp micro-homology arms via CRISPR/Cas9 system. The donor DNAs of MHi-pyrG1-hph and MHi-kusA-hph were co-transformed with linear sgRNA constructs (sgRNA-pyrG1 and sgRNA-kusA) and Cas9 expression plasmid pCas9-hyh into the protoplasts of *A. niger* D and D353. Two DSBs were generated by the Cas9 under the guide of sgRNA, and then were repaired by HR with the integration of donor DNAs. **b**, **c** Diagnostic PCR analysis of the selected *pyrG* deficient transformants. The expected sizes of PCR products of the mutants were 3240-bp (pyrG-g-F/pyrG-g-R1) and 2702-bp (kusA-g-F/kusA-g-R), when the selection marker *hph* cassette were correctly inserted at the loci of *pyrG* or *kusA*. The expected sizes of PCR products of the hosts were 1345-bp (pyrG-g-F/pyrG-g-R1) and 804-bp (kusA-g-F/kusA-g-R), when the hph markers were not inserted at the loci of *pyrG* or *kusA*. For *A. niger* D353, the PCR products of some mutants using pyrG-g-F/pyrG-g-R1 were smaller than the expected size. After sequencing theses PCR products, it’s found that the smaller inserted fragments were the sgRNA-pyrG1 expression cassette

To establish genetic manipulation of citric acid producing isolates WT-D and D353, we chose the traditional selection marker *pyrG* gene and key gene *kusA* involved in NHEJ system as targets [10]. To simultaneously disrupt these two targets, two double strand breaks were introduced at respective loci using Cas9 with corresponding guides and then were repaired by homologous recombination system (Fig. 1). For each strain, 24 primary transformants were randomly picked and subcultured onto either MM agar, MM agar supplemented with uridine, and MM supplemented with 5′-FOA, in order to screen the *pyrG* deficient mutants. As shown in Additional file 1: Figure. S1, *pyrG* deficient mutants were able to grow on the MM with uridine and MM with 5′-FOA, but can’t grow on the MM plate without uridine. For both WT-D and D353, 11 *pyrG* deficient mutants among 24 detected transformants were selected for further PCR verification. The genomes of these *pyrG* deficient transformants were extracted and verified with the corresponding primer pairs (Fig. 1). For *A. niger* WT-D, two mutants D10 and D20 were confirmed as the *pyrG/kusA* double mutants, while for *A. niger* D353, D353.8 was verified as the *pyrG/kusA* mutant.

To test whether the *pyrG* and *kusA* deficient mutants can be used for further genome editing, the *A. niger* D10 and D353.8 were selected as progenitor strains for disruption of the polyketide synthase encoding gene *albA*, which is required for synthesis of DHN melanin and conidial pigmentation. With the selection for *pyrG* integration into the genomes, all the primary transformants were confirmed to be prototrophic, which can be grow normally on the MM agar without uridine. Due to the high efficiency of CRIPSR/Cas9 and high frequency of homologous recombination in the NHEJ system deficient strains, all the selected transformants were showed demonstrated to be genome edited (Additional file 1:Figure S2).

We simultaneously disrupted both *pyrG* and *kusA* in two citric acid producing strains (Fig. 1) and constructed the fungal chassis which the further recyclable genome editing (Additional file 1: Figure S2). The results here was consistent with the previous study reported in *Aspergillus orzye*, in which the *pyrG* gene was also disrupted with the other important gene involved in NHEJ, the *ligD* gene, to generate the mutant with highly efficient gene targeting [29]. Taken together, it demonstrated that the *pyrG* and *kusA* disrupted mutants could be as utilized as citric acid producing chassis to facilitate the recyclable genome editing.

### Gene disruption of *pyrG* significantly elevated citric acid titers in shake flaks culture

**Fig. 2:**
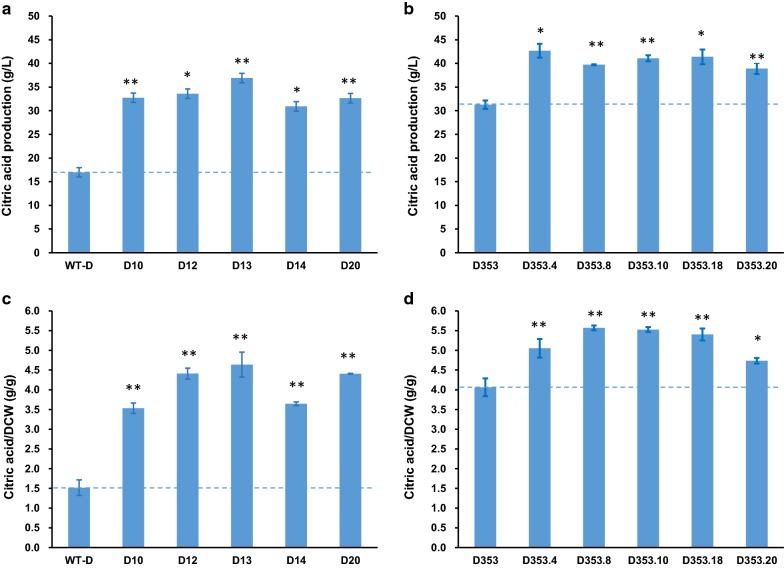
Citric acid fermentation characteristics of the *pyrG* disrupted mutants. **a**, **b** citric acid titer and **c**, **d** normalized citric acid titer (g citric acid/g dry weight) were calculated for each strain. 1 × 10^5^ spores/mL were inoculated in 20 mL citrate fermentation (CitFM) media and incubated at 34 °C for 96 h. The extracellular citric acid was determined by the method of HPLC. WT-D and D353, the parent strains used as the control; D10, D12, D13, D14, and D20, the *pyrG* deficient mutants derived from WT-D; D353.4, D353.8, D353.10, D353.18, D353.20, the *pyrG* deficient mutants derived from D353. Among them, D10, D20 and D353.8 were the *pyrG* and *kusA* double deficient mutants. Pairwise Student’s t-tests were conducted between conditional expression mutant relative to the parent strains. *p* values are indicated as (< 0.05, *; < 0.01, **; < 0.001, ***)

During routine quality control of transformant isolates and progenitor strains, we observed an unexpected increase of citric acid titers in the culture supernatants for mutants of both WT-D and D353. To confirm this observation, five *pyrG* deficient mutants derived from either *A. niger* WT-D and D353 were selected for citric acid fermentation in shake flasks. This assay demonstrated that significantly increased citric acid titers were observed for all the *pyrG* deficient mutants (Fig. 2). For *pyrG* mutants derived from *A. niger* WT-D, citric acid increased from 1.92-fold (D14, 30.94 ± 0.58 g/L) to 2.17-fold (D13, 33.59 ± 3.24 g/L), compare to the progenitor control strain (WT-D, 16.98 ± 1.91 g/L) (Fig. 2a). Additionally, mutants derived from *A. niger* D353 also demonstrated increased citric acid titers from 1.24-fold (D353.20, 38.92 ± 1.17 g/L) to 1.36-fold (D353.4, 42.69 ± 1.48 g/L), compared to control (31.28 ± 0.88 g/L) (Fig. 2b). Normalized citric acid titers to biomass of all isolates confirmed increased citric acid production in mutant strains (Fig. 2c, d). For the *pyrG/kusA* double mutants, D10, D20 and D353.8 showed the similar citric acid production as these *pyrG* single mutants did, and the *kusA* single mutants did not influence the citric acid titers (data not shown). While it can be noted that the increase in citric acid titers following *pyrG* deletion was greater in isolate WT-D when compared to D353 (Fig. 2), magnitude of increased citric acid titer was sufficiently high in both backgrounds to warrant further investigation. Thus, we hypothesized that *pyrG* deletion may increase citric acid production in *A. niger*.

### Generation of a *pyrG* conditional expression mutant in *A. niger*

**Fig. 3:**
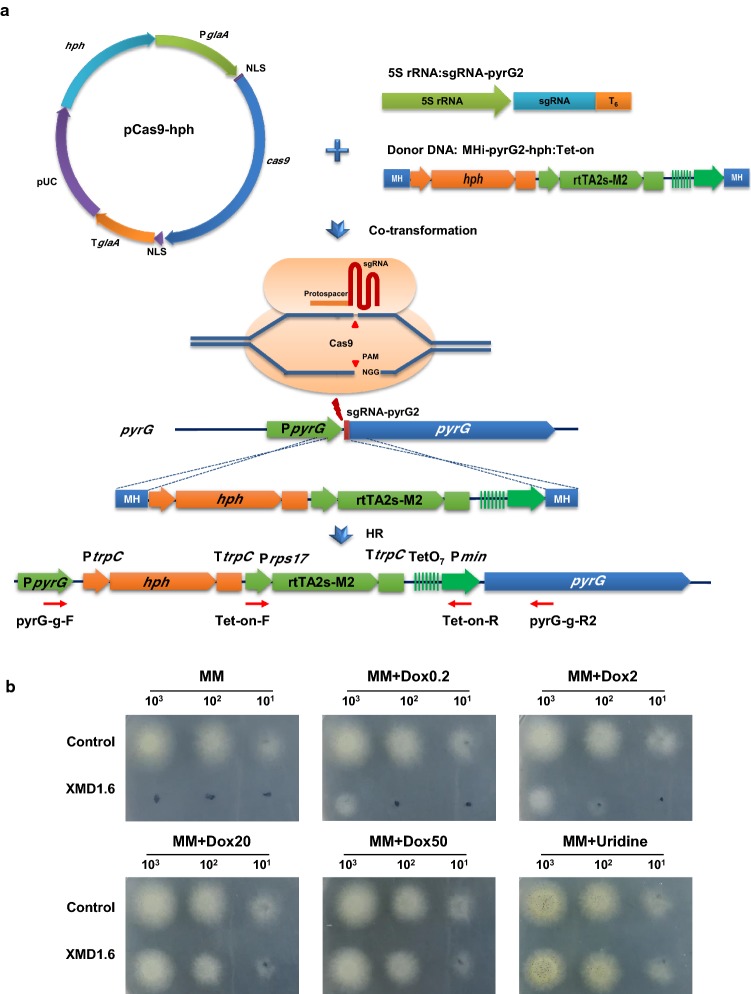
Titratable expression of *pyrG* mutagenesis constructed in *A. niger*. **a** Schematic diagram of *pyrG* titratalbe expression mutagenesis mediated by integrating the donor DNA with 40-bp micro-homology arms via CRISPR/Cas9 system based 5S rRNA. The donor DNA MHi-pyrG2-hyh:Tet-on, containing the Tet-on cassette, were co-transformed with linear sgRNA construct sgRNA-pyrG2 and Cas9 expression plasmid pCas9-hyh into the protoplasts of *A. niger* D353. DSBs at the locus of the upstream of *pyrG* encoding sequences, were generated by the Cas9 under the guide of sgRNA-pyrG2, and then were repaired by HR with the integration of donor DNA MHi-pyrG2-hyh:Tet-on, resulting in the replacement of *pyrG* native promoter. **b** Phenotypic screening of pyrG conditional expression mutants on solid plates. 1 × 10^3^, 1 × 10^2^, and 1 × 10^1^ spores were inoculated in 2 µL volumes onto the MM supplemented with various concentrations of Dox and MM with uridine as control. Plates were incubated at 30 °C in the dark for 48 h. Representative images are shown for technically triplicated experiments. Control, *A. niger* D353 as the positive controls; XMD1.6, the *pyrG* conditional expression mutants

The *pyrG* gene encoding orotidine-5′-monophosphate (OMP) decarboxylase plays an essential role in uridine biosynthesis [21], but there is no research to unveil its impact on the other cell metabolism. To further investigate the influence of *pyrG* expression on citric acid production, we constructed the conditional expressed mutant of *pyrG* by in situ integration of the Tet-on system at the upstream of *pyrG* encoding sequences (Fig. 3). Consistent with the previous studies [31–34], Tet-on system is a useful method to functionally analyze essential genes, enabling analyses of null, loss-of-function, and over-expression in a single strain by addition of the inducer Dox. After co-transformation, ten randomly selected primary transformants were subcultured on MM plates, MM supplemented with 20 µg/mL Dox and MM supplemented with uridine. Six transformants were able to grow on MM with uridine but were unable to grow on MM plate, suggesting that these transformants were *pyrG* deficient mutants. Among these mutants, only two transformants resembled to the *pyrG*^+^ phenotype when 20 µg/mL Dox was supplemented into the MM plate. After verifying these genotypes via genomic PCR diagnosis (Additional file 1: Figure S3), isolate XMD1.6 was used as the conditional expression mutant for the further experiments.

In order to confirm titratable *pyrG* expression in isolate XMD1.6, phenotypic screens of the conditional mutant were conducted on MM solid agar supplemented with 0, 0.2, 2, 20 and 50 µg/mL Dox, respectively. No growth of the conditional expression mutant was observed in the absence of Dox in growth media, and the mutant was indistinguishable from the control on the MM plate with uridine but without Dox (Fig. 3). However, the titration of Dox in MM agar without uridine enabled isolate XMD1.6 to prototrophy, and ultimately to generate colonies which resembled the parental strain. These data confirm Dox dependent titration of *pyrG* expression in the XMD1.6 mutant.

### Reduced *pyrG* expression improves citric acid titers in *A. niger* submerged culture

**Fig. 4:**
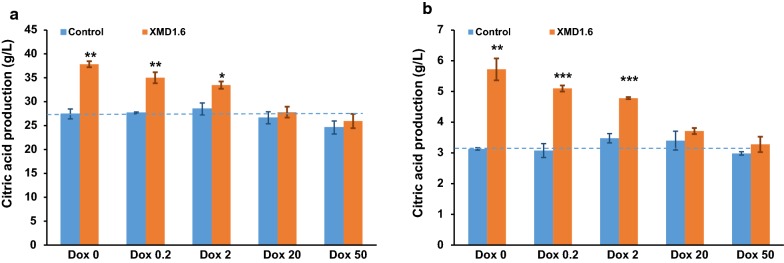
Citric acid fermentation characteristics of *pyrG* titratable expression mutant. **a** Citric acid titer and **b** normalized citric acid titer (g citric acid/g dry weight) was detected and calculated for each Dox concentration. 1 × 10^5^ spores/mL were inoculated in 20 mL citrate fermentation (CitFM) media with different Dox concentration and incubated at 34 °C in the dark for 96 h. The extracellular citric acid was determined by the method of HPLC. Pairwise Student’s t-tests were conducted between conditional expression mutant relative to the parent strains. *p* values are indicated as (< 0.05, *; < 0.01, **; < 0.001, ***)

**Fig. 5:**
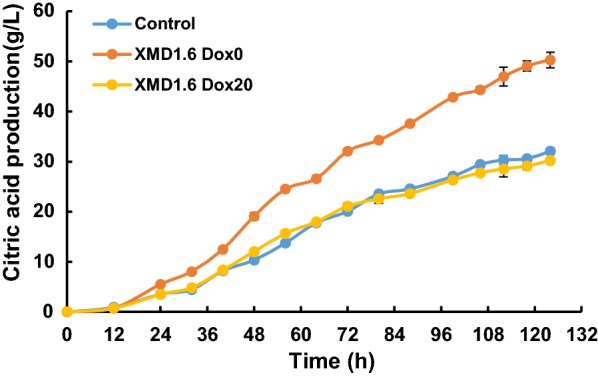
Citric acid production of *pyrG* titratable expression mutant in 5 L bioreactor. Citric acid titers of *pyrG* titratable expression mutant under 0 and 20 μg/mL Dox were compared with its parent strains in the 5 L bioreactor. 1 × 10^5^ spores/mL were inoculated in 3 L citrate fermentation (CitFM) media at 34 °C in the dark for 124 h. The extracellular citric acid was determined by the method of HPLC. Control, the parent strains D353; XMD1.6, the *pyrG* conditional expression mutants

In order to test the influence of *pyrG* expression levels on citric acid production, we conducted citric acid fermentation with various Dox concentrations using *pyrG* conditional expression mutant XMD1.6. As shown in Fig. 4, *pyrG* expression significantly influenced the citric acid production of *A. niger*, with titers and proportion of total acid gradually reduced with the increase of the Dox supplementation into fermentation media. These data further suggest that PyrG has a negative impact on in *A. niger* citric acid production. Without Dox or with 0.2 μg/mL Dox supplemented, the citric acid production of *pyrG* conditional expressed mutants XMD1.6 increased about 1.38-fold (37.8 ± 0.63 g/L) and 1.27-fold (35.02 ± 1.16 g/L) as that of the control D353 (27.43 ± 1.03 g/L) respectively, which is similar to the results of *pyrG* disrupted mutants (Fig. 2). With 2 μg/mL Dox supplemented, the citric acid production of *pyrG* conditional expressed mutants XMD1.6 slightly increased about 1.17-fold (33.5 ± 0.76 g/L) as that of the control strain (Fig. 4). It should be noted that *pyrG* null or low expression also reduced the cell growth. However, normalization of citric acid titers against biomass of XMD1.6 confirmed 1.83-fold, 1.66-fold, and 1.38-fold increased citric acid titers without or with 0.2 and 2 μg/mL Dox supplemented, compared to that of the D353 control. In contrast, when 20 or 50 µg/mL Dox was supplemented to growth media, the citric acid production of XMD1.6 showed no statistically significant difference compared to the parent strain D353 (Fig. 4). The similar results were also obtained when citric acid fermentations were carried out in 5 L bioreactor (Fig. 5). Without the Dox supplemented, the final citric acid production of XMD1.6 reached up to 50.30 ± 1.55 g/L, with the max citric acid productivity of 1.00 g/L/h, which increase about 1.57-fold as to the parent strain (32.08 ± 0.59 g/L). In contrast, when 20 µg/mL Dox supplemented, the citric acid titer of XMD1.6 (30.23 ± 0.52 g/L) resembled to the parent strain. As to by-products, only a small amount of oxalic acid and succinic acid were detected in the end-point samples, while acetic acid and fumaric acid were not detected (Additional file 1: Figure S4). For instance, without the Dox supplemented, the final titer of oxalic acid and succinic acid of XMD1.6 were 1.35 ± 0.07 g/L and 2.16 ± 0.05 g/L, respectively. These data therefore support the hypothesis that citric acid production improved due to the disruption or down-regulated expression of *pyrG*.

### Metabolite profile analysis of *A. niger* D353 and *pyrG* disrupted mutant

**Fig. 6:**
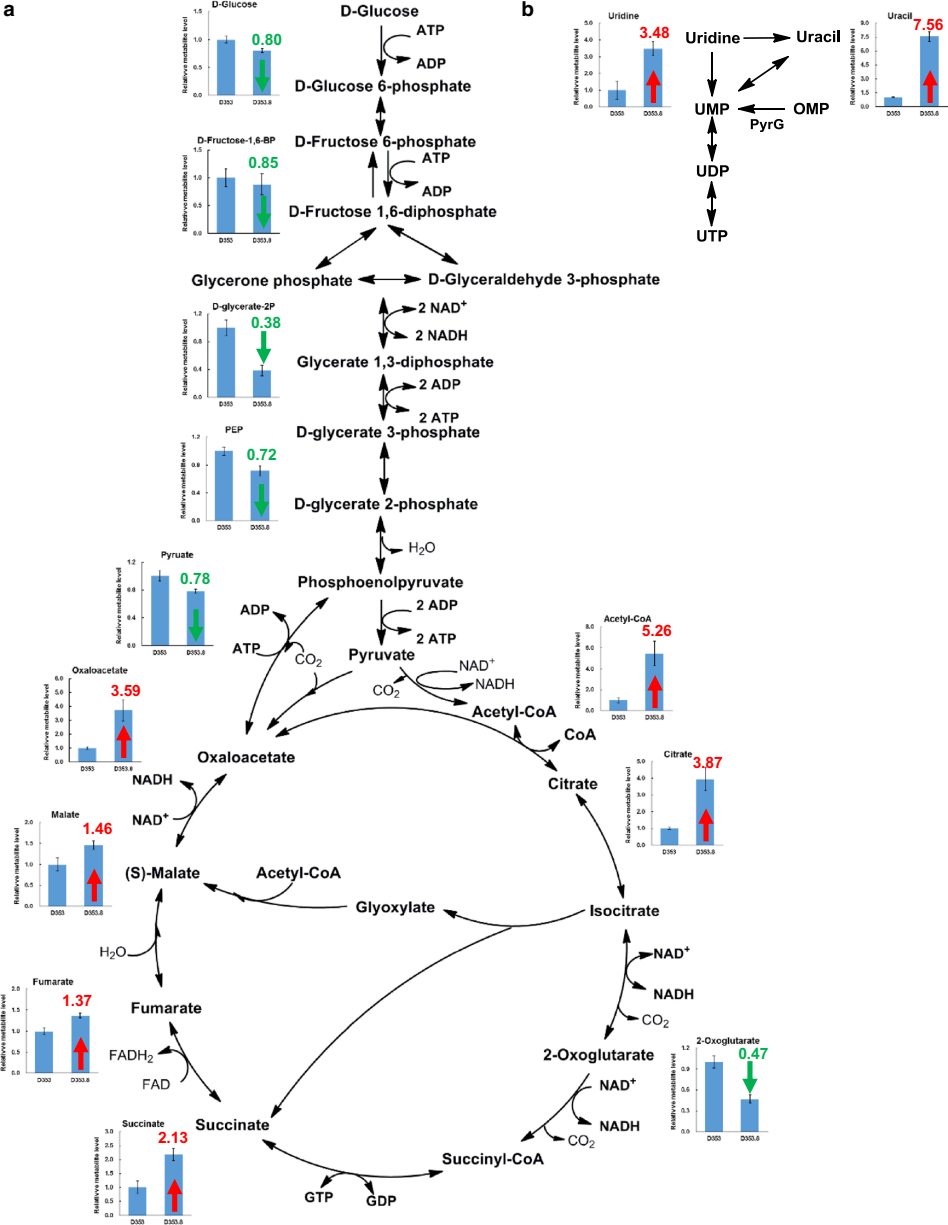
Intracellular metabolite profiling of the *A. niger* D353 and *pyrG* mutant D353.8. Intracellular metabolite profiling of the intermediates involved in central metabolism (**a**) and pyrimidine biosynthesis pathway (**b**). The relative level of each metabolite in *A. niger* D353.8 was calculated by dividing its normalized peak area by that of the control D353. Red arrows represent the relative quantitative change of the metabolite increase in *A. niger* D353.8, while green arrows represent the relative quantitative change of the metabolite decrease. The number next to the arrow represented the fold change of each intracellular metabolite in *A. niger* D353.8 against the ones in D353. *OMP* orotidine 5′-phosphate, *UMP* uridine monophosphate, *UDP* uridine biphosphate, *UTP* uridine triphosphate

In order to elucidate the impact of *pyrG* disruption on the *A. niger* intracellular metabolite profile, metabolomics analyses of parent strain D353 and *pyrG* deficient mutant D353.8 were conducted by our established LC–MS/MS pipeline [35]. Samples were taken at the end of citric acid fermentation in shake flasks (Fig. 6). Metabolite analysis demonstrated that most of the Embden–Meyerhof pathway (EMP) intermediates decreased in concentration, while citrate and its precursor Actyl-CoA and oxaloacetate significantly increased in the *pyrG* mutant when compared to the control. With regard of TCA cycle intermediates, 2-oxoglutarate decreased significantly in the mutant, but the malate, fumarate and succinate increased in different degrees (Fig. 6). The data suggest that *pyrG* disruption dramatically disturbed the intracellular central metabolism and improved the intracellular level of the citric acid and its precursor, which may lead to the extracellular citric acid accumulation. Considering the EMP intermediates decreased while the citric acid precursors acetyl-CoA and oxaloacetate were significantly increased, it suggested that this metabolic change may be caused by the accelerated glycolysis pathway and the insufficient activity of the citrate synthetase. In *Aspergillus nidulans*, it is reported that the genes involved in carbon metabolism, such as α-glucosidase B (*agdB*) and sugar transporter, were significantly up-regulated in a *pyrG* deleted mutant [36], which is consistent with the EMP metabolism changes in this study. Therefore, we speculated that higher citrate synthetase expression could further improve citric acid production when *pyrG* expression was disrupted or reduced.

What’s more, the intermediates in the uridine pathway were also observed. PyrG is essential for the de novo pyrimidine biosynthesis, which catalyzes the carboxylation of OMP into uridine monophosphate (UMP) [21, 36]. When the *pyrG* gene is deleted, the UMP is synthesized by the salvage pathway from uridine or uracil [36]. As shown in Fig. 6, compared to the control, the intracellular uracil and uridine increased 7.56-fold and 3.48-fold in the *pyrG* mutant, respectively, while the intracellular OMP were not detected. Sun et al. also observed the similar phenomenon that the *pyrG* mutation leads to uracil and uridine accumulation in the cells of *A. nidulans* [36]. These metabolite profiling data indicated that the *pyrG* deletion may influence the global metabolic network. Future studies, such as dynamic metabolomic and transcriptomic experiments, will be carried out to fully elucidate the molecular basis about the impact of the *pyrG* gene on citric acid production.

## Conclusions

In this study, we demonstrate that *pyrG* impacts organic acid metabolism in *A. niger* leading to potential industrial benefits. Both gene disruption and down-regulation of *pyrG* significantly increases of citric acid production in *A. niger*. We suggest that *pyrG* is a promising target for metabolic engineering of filamentous fungal cell factories.

## Methods

### Strains and cultivation conditions

The strains used in this study are listed in Table 1. The citric acid producing strains *A. niger* WT-D and D353 were purchased from Shanghai Industrial Microbiology Institute Tech. Co. (Shanghai, China), Ltd. The wild-type *A. niger* WT-D was isolated on acid PDA media from soil samples, and the D353 was derived from WT-D after Co^60^ γ- ray radiation mutagenesis. *Escherichia coli* DH5α (Transgene, Beijing, China) was used for plasmid construction and cultured at 37 °C in Luria–Bertani broth containing ampicillin (100 μg/mL). *A. niger* strains were cultivated on defined minimal medium (MM) as reported previously [22], or on complete medium (CM) consisting of MM supplemented with 0.5% yeast extract and 0.1% casamino acids. 1.5% agar was supplemented for plates. When necessary, 150 μg/mL of hygromycin was added for the *hph* selection marker; 0.75% 5-fluoroorotic acid (5-FOA) and 10 mM uracil were used in the MM for the *pyrG* mutants.

**Table 1:** *S*trains and plasmids used in the study

Name	Description	References
WT-D	Citric acid producing strain isolated from soil sample	Lab store
D353	Derived from WT-D after Co60 γ- ray radiation mutagenesis	Lab store
D10	*kusA::hph*, *pyrG::hph*, *hyg*^*R*^	This study
D12	*pyrG::hph*, *hyg*^*R*^	This study
D13	*pyrG::hph*, *hyg*^*R*^	This study
D14	*pyrG::hph*, *hyg*^*R*^	This study
D20	*pyrG::hph*, *hyg*^*R*^	This study
D353.4	*pyrG::hph*, *hyg*^*R*^	This study
D353.8	*kusA::hph*, *pyrG::hph*, *hyg*^*R*^	This study
D353.10	*pyrG::hph*, *hyg*^*R*^	This study
D353.18	*pyrG::hph*, *hyg*^*R*^	This study
D353.20	*pyrG::hph*, *hyg*^*R*^	This study
XMD1.6	*PpyrG::Tet*-*on*, *hyg*^*R*^	This study
pSM-AnpyrG	PtrpC:*AnpyrG*:TtrpC	This study
pCas9-hph	P*glaA*:nls-Cas9-nls:T*glaA, hyg*^*R*^	This study
pCas9-AnpyrG	P*glaA*:nls-Cas9-nls:T*glaA, pyrG*^*R*^	This study
psgRNA6.1	P*5S rRNA*:sgRNA-albA:T*poly(T)*_*6*_	[10]
psgRNA6.13	P*5S rRNA*:sgRNA-kusA:T*poly(T)*_*6*_	This study
psgRNA6.14	P*5S rRNA*:sgRNA-pyrG1:T*poly(T)*_*6*_	This study
psgRNA6.15	P*5S rRNA*:sgRNA-pyrG2:T*poly(T)*_*6*_	This study

### Genetic manipulation

The plasmids used in this study were listed in Table 1. The protospacers and primers were listed in Additional file 1: Tables S1, S2, respectively. For recycling genome editing using the selection marker *pyrG*, the *pyrG* coding sequence was amplified with the *A. nidulans* genome as template and primers AnpyrG-F and AnpyrG-R, then cloned into the backbone of reverse amplified using pSilent-1 as temple and primers Ptrp-Rrev and TtrpC-Frev using the ClonExpress™ one step cloning kit (Vazyme, C113), resulting in pSM-AnpyrG. To construct the Cas9 expression plasmid hygromycin resistance, the selection marker *hph* cassette was amplified with the template pSilent-1 [37] and the primer pair of PtrpC-Fm and TtrpC-Rm. The plasmid backbone was amplified using pCas9 as the template with the primer pair of pGm-Frev and pGm-Rrev. The *hph* expression cassette and the *AnpyrG* expression cassette were then cloned into the plasmid backbond via the ClonExpress™ one step cloning kit (Vazyme, C113), resulting in pCas9-hph and pCas9-AnpyrG, respectively (Table 1).

To construct the sgRNA targeting different genes, protospacers were predicted sgRNA by the sgRNAcas9 software [38], and designed with the minimal off-target possibility. The targeting sgRNA constructs were built by digestion of sgRNA expression plasmids psgRNA6.0 [10] with *Bbs*I, and ligation with synthetic double stranded oligonucleotides of the desired sequences. The linear sgRNA targeting expression cassettes with 5S rRNA gene as promoter for *A. niger* transformation were obtained by amplification using sequence verified corresponding plasmids as template and primers M13F and M13R as previously described [10]. The DNA sequences of sgRNA constructs are given in Additional file 1: Table S3.

The donor DNAs with micro-homology (40-bp) flanks were generated as previously described [10]. These micro-homology sequences were designed adjacent to the 5′ and 3′ regions of the target sequence without the PAM site. The linear donor DNA constructs MHi-kusA-hph and MHi-pyrG1-hyh for homologous recombination of selection marker *hph* into the *kusA* and *pyrG* were generated by PCR using pSilent-1 [37] as template and primers MHi-kusA-Fm/MHi-kusA-Rm and MHi-pyrG-Fm/MHi-pyrG-Rm, respectively. Similarly, the donor DNA MHi-albA-AnpyrG was amplified with pSM-AnpyrG as template and the primers MHi-albA-Fm/MHi-albA-Rm. The linear donor DNA MHi-pyrG2-hyh:Tet-on was generated by PCR with the template pTC1.13 and the primer pair MHi-pyrG2-Fm/MHi-pyrG2-Rm. After purification by PCR products purification kit, PCR products were used for *A. niger* transformation. DNA sequences of donor DNA are given in Additional file 1: Table S4.

### DNA transformation

The standard protocol of *A. niger* genome editing using the CRISPR/Cas9 system based 5S rRNA was performed as previously described [10]. For simultaneous gene insertion of *kusA* and *pyrG*, 2 μg the donor DNA constructs MHi-kusA-hph and MHi-pyrG1-hyh as HR template were co-transformed into the protoplasts of *A. niger* D and D353 together with their corresponding sgRNA targeting constructs sgRNA-kusA and sgRNA-pyrG1 and the pCas9-hyh. For gene insertion of *albA*, 2 μg sgRNA-albA constructs and 2 μg donor DNA constructs MHi-albA-ANpyrG into the protoplasts of *kusA*/*pyrG* double mutants *A. niger* D.10 and D353.8, respectively. For the *pyrG* titratible expression mutants, the donor DNA MHi-pyrG2-hyh:Tet-on, containing the Tet-on cassette, were co-transformed with linear sgRNA construct sgRNA-pyrG2 and Cas9 expression plasmid pCas9-hyh into the protoplasts of *A. niger* D353. After twice subculture and purification, genomic DNA of random selected transformants was extracted and verified via diagnostic PCR and sequencing analysis with the corresponding primers.

### Phenotypic analysis on solid media

To analyse the phenotype of *pyrG* mutants, *A. niger* conidia were harvested in 0.9% NaCl solution from 5-day cultivated CM agar plates. Spores of *A. niger* isolates were spotted with 2 µl at the contraction of 10^5^/mL on MM agar plates without uridine, with uridine and with uridine and 5-FOA, which were incubated for 4 days at 30 °C. For the *pyrG* titratable expression mutants, 1 × 10^3^, 1 × 10^2^, and 1 × 10^1^ spores were inoculated in 2 µl volumes onto the MM supplemented with various concentrations of Dox and MM with uridine as control. Plates were incubated at 30 °C in the dark for 48 h. Phenotypic detections were conducted in technical triplicate.

### Citric acid fermentation

Citrate fermentation was carried out using the liquefied corn media [35]. The final concentration of 1 × 10^5^ spores/mL was inoculated in 20 mL liquefied corn media with different concentration of Dox in 100 mL shake flasks, which were cultivated at 34 °C and 220 rpm for 96 h. The weight of the shake flasks were measured before and after the citric acid fermentation to eliminate measurement errors caused by evaporation. For the citric acid production in the 5 L bioreactor with a stirring paddle device, most identical fermentation parameters were utilized for 124 h, but the aeration rate was coupled to dissolved oxygen concentration (> 60%).

Supernatants were filtered from cultures using filter paper. Total acids were first titrated using 0.1429 M NaOH with 20 μL 0.1% phenolphthalein as pH indicator. Next, the supernatants were diluted in sterile distilled water depending on the estimated total acid. Samples were boiled for 15 min at 100 °C, after which supernatants were centrifuged at 12,000 rpm for 5 min and filtered through a 0.22 μm sterile filter membrane. Extracellular organic acids were detected by Prominence UFLC equipped with a UV detector (Shimadzu, Kyoto, Japan) and a Bio-Rad Aminex HPX-87H column (300 × 7.8 mm) according to the procedure described previously.

### Intracellular metabolite analysis by LC–MS/MS

The samples for intracellular metabolite profiling were prepared and detected based on the standardized and improved LC–MS/MS metabolomics methodology [35]. Briefly, 5 mL (± 0.5) of *A. niger* mycelial culture was fast filtered by a -20 °C pre-cooled vacuum filter with six layers Miracloth (CalBiochem, Merck, Darmstadt, Germany). After washing with 25 mL pro-cooled PBS buffer, the mycelial samples were flash frozen into liquid nitrogen. After pulverization by pestle and mortar in liquid nitrogen, about 0.1 g (± 0.01) of sample (equal to about 10 mg dry weight) was suspended in 1 mL pre-heated 75% ethanol and incubated at 100 °C for 15 min. Then, the mixture was centrifuged at 12,000 rpm for 5 min at 0 °C and the supernatant was collected into a precooled tube. The cell pellets were re-suspended with another 1 mL of extraction solution and processed by boiling-water bath for 15 min. The second mixture was centrifuged again and the supernatant extraction were mixed with the first supernatant and centrifuged at 12,000 rpm for 30 min at 0 °C. The supernatant was then collected, freeze dried and detected by the LC–MS/MS platform containing Ultra-performance liquid chromatography (UPLC) 30A (Shimadzu, Kyoto, Japan) and TripleTOF™ 6600 mass spectrometer (Applied Biosystem Sciex, USA) as described previously [35]. The LC–MS/MS data were normalized by sample weight, and then metabolites involved in central metabolism were identified according to the protocol described [35].

### Determination of fungal biomass

To determine fungal biomass after citric acid fermentation, cultures were vacuum filtered through filter paper, washed in 5-fold sterile water, and added to pre-weighed falcon tubes. Biomass was incubated at 50 °C until dry (minimum of 24 h), after which dry weight was determined.

## Supplementary information

**Additional file 1: Figure S1.** Phenotypic comparison of *pyrG* and *kusA* mutants derived by *A. niger* D and D353. **Figure S2.** Insertion inactivation of the *albA* gene in *A. niger* D10 and D353.8. **Figure S3.** Titratable expression of *pyrG* mutagenesis constructed in *A. niger*. **Figure S4.** Extracellular organic acids of *pyrG* titratable expression mutant in 5 L bioreactor. **Table S1.** Protospacers used in this study, **Table S2.** Primers used in this study. **Table S3.** DNA sequences of sgRNA constructs used in this study. **Table S4.** DNA sequences of donor DNAs used in this study.

## Data Availability

All data generated or analyzed during this study are included in this published article and its additional files.
