# Supplementary material for: Disruption or reduced expression of the orotidine-5′-decarboxylase gene pyrG increases citric acid production: a new discovery during recyclable genome editing in Aspergillus niger
Source: Microb Cell Fact. 2020 Mar 24;19:76. doi: 10.1186/s12934-020-01334-z (PMC7092557; doi:10.1186/s12934-020-01334-z)
Supplement: Supplementary file 1 — Additional file 1: Figure S1. Phenotypic comparison of pyrG and kusA mutants derived by A. niger D and D353. Figure S2. Insertion inactivation of the albA gene in A. niger D10 and D353.8. Figure S3. Titratable expression of pyrG mutagenesis constructed in A. niger. Figure S4. Extracellular organic acids of pyrG titratable expression mutant in 5 L bioreactor. Table S1. Protospacers used in this study, Table S2. Primers used in this study. Table S3. DNA sequences of sgRNA constructs used in this study. Table S4. DNA sequences of donor DNAs used in this study. [file 12934_2020_1334_MOESM1_ESM.docx]

Additional information

**Disruption or reduced expression of the orotidine-5'-decarboxylase gene *pyrG* increases citric acid production in *Aspergillus niger***

Lihui Zhang^a1,2,3†^, Xiaomei Zheng^2,3,4†^, Timothy C. Cairns^2,3^, Zhidan Zhang^2^, Depei Wang^1*^, Ping Zheng^2,3,4*^, Jibin Sun^2,3,4^

^1^ College of Biotechnology, Tianjin University of Science & Technology, Tianjin, 300457, China

^2^ Tianjin Institute of Industrial Biotechnology, Chinese Academy of Sciences, Tianjin, 300308, China

^3^ Key Laboratory of Systems Microbial Biotechnology, Chinese Academy of Sciences, Tianjin, 300308, China

^4^ University of Chinese Academy of Sciences, Beijing, 100049, China

**^*^Correspondence:**

**Ping Zheng**, Tel: +86-22-84861945, Fax: +86-22-84861943 E-mail: [zheng_p@tib.cas.cn](mailto:zheng_p@tib.cas.cn),

**Depei Wang**, E-mail: wangdp@tust.edu.cn, Tel/Fax: 86-22-60600127

^†^Lihui Zhang and Xiaomei Zheng contributed equally to this work.

**E-mail addresses:**

Lihui Zhang: zhanglh@tib.cas.cn

Xiaomei Zheng: [zheng_xm@tib.cas.cn](mailto:zheng_xm@tib.cas.cn)

Timothy C. Cairns: cairns@tib.cas.cn

Zhidan Zhang: zhang_zd@tib.cas.cn

Depei Wang: wangdp@tust.edu.cn

Ping Zheng: [zheng_p@tib.cas.cn](mailto:zheng_p@tib.cas.cn)

Jibin Sun: sun_jb@tib.cas.cn

**
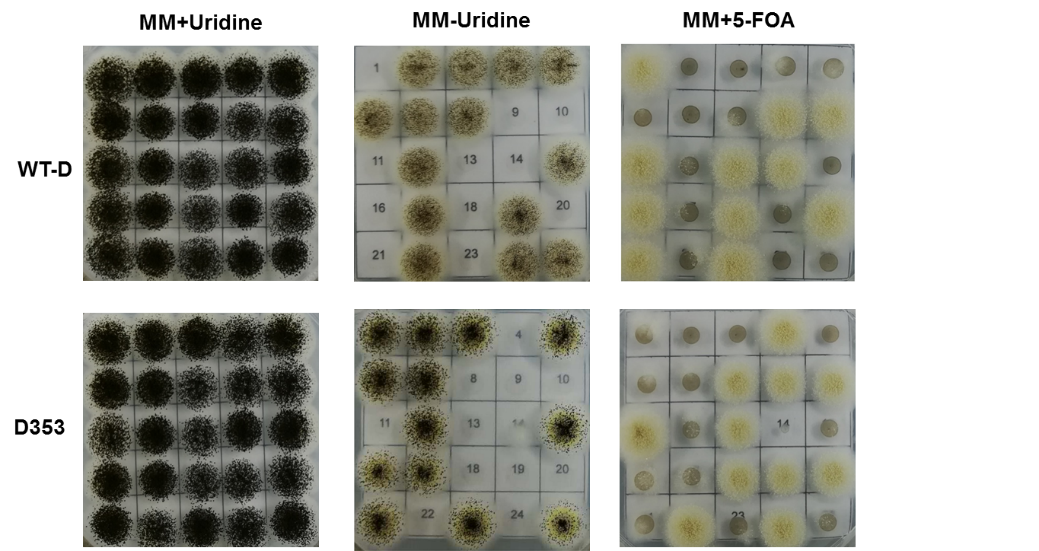
**

Figure S1 Phenotypic comparison of *pyrG* and *kusA* mutants derived by *A. niger* D and D353

Phenotypic comparison of the randomly selected 24 transformants derived from WT-D and D353. 1 × 10^4^ spores were inoculated in 2 µl volumes onto the MM and MM supplemented with uridine and 5-FOA and MM with uridine. Plates were incubated at 34°C for 3 days. Representative images are shown for technically triplicated experiments. The *pyrG* disrupted mutants enable to grow on the MM supplemented with uridine and 5-FOA, but can’t grow on MM without uridine.


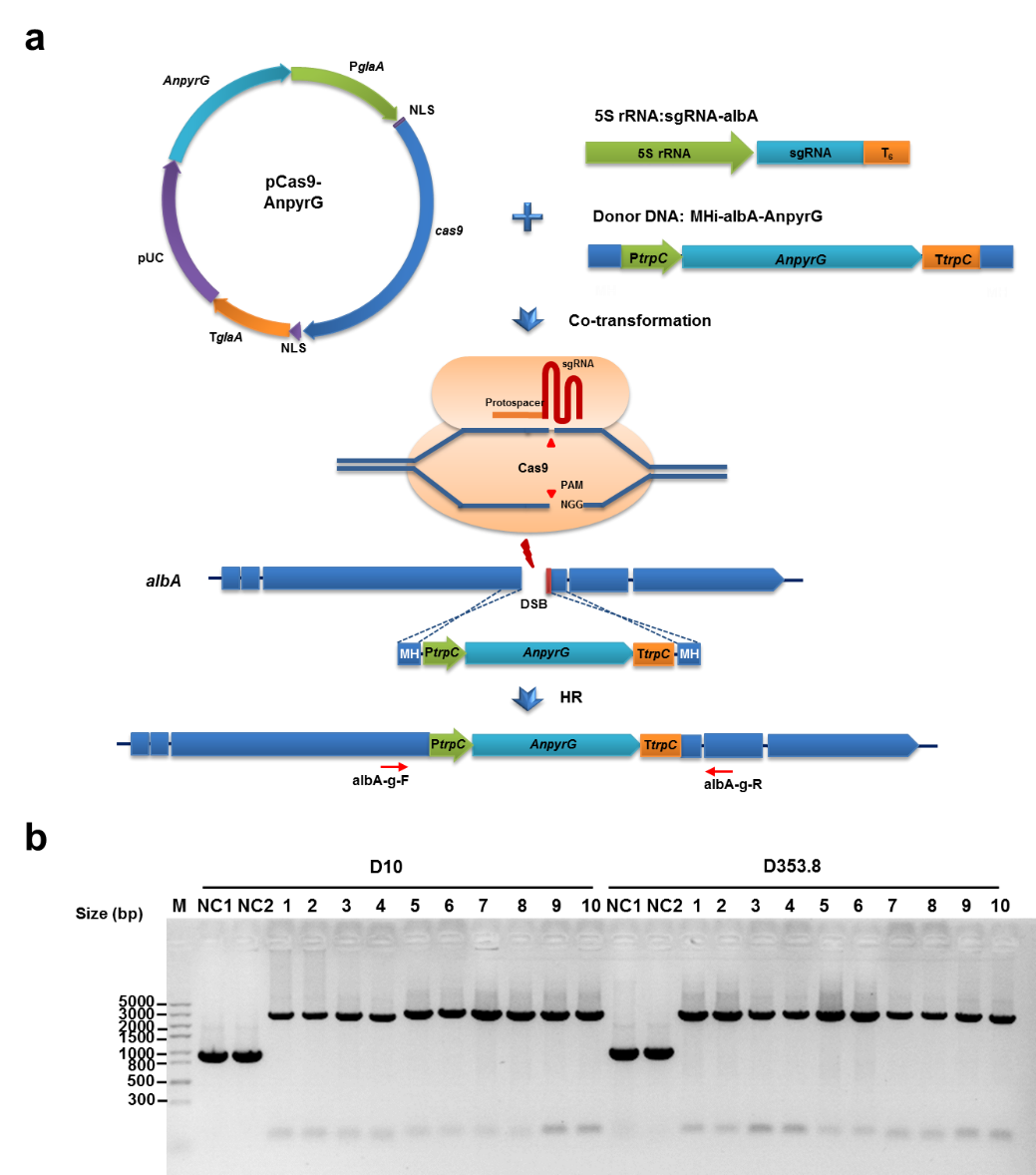


Figure S2 Insertion inactivation of the *albA* gene in *A. niger* D10 and D353.8

(a) Schematic diagram of disrupted mutagenesis of *albA* gene mediated by integrating the donor DNA with 40-bp micro-homology arms. The donor DNA MHi-albA-AnpyrG was co-transformed with linear sgRNA constructs (sgRNA-albA) and Cas9 expressing plasmid pCas9-AnpyrG into the wild type *A. niger* D10 and D353.8. DSBs were generated by the Cas9 under the guide of sgRNA, and then were repaired by HR with the integration of MHi-albA-AnpyrG. (b) Diagnostic PCR analysis of 10 selected transformants. The expected sizes of PCR products were 2608-bp (albA-g-F/albA-g-R), when the *hph* marker was correctly inserted at the *albA* locus. Negative control NC1 only co-transformed with pCas9-AnpyrG and donor DNA MHi-albA-AnpyrG. Negative control NC2 co-transformed with pCas9-AnpyrG, scrambled sgRNA (sgRNA-s) and donor DNA MHi-albA-AnpyrG.


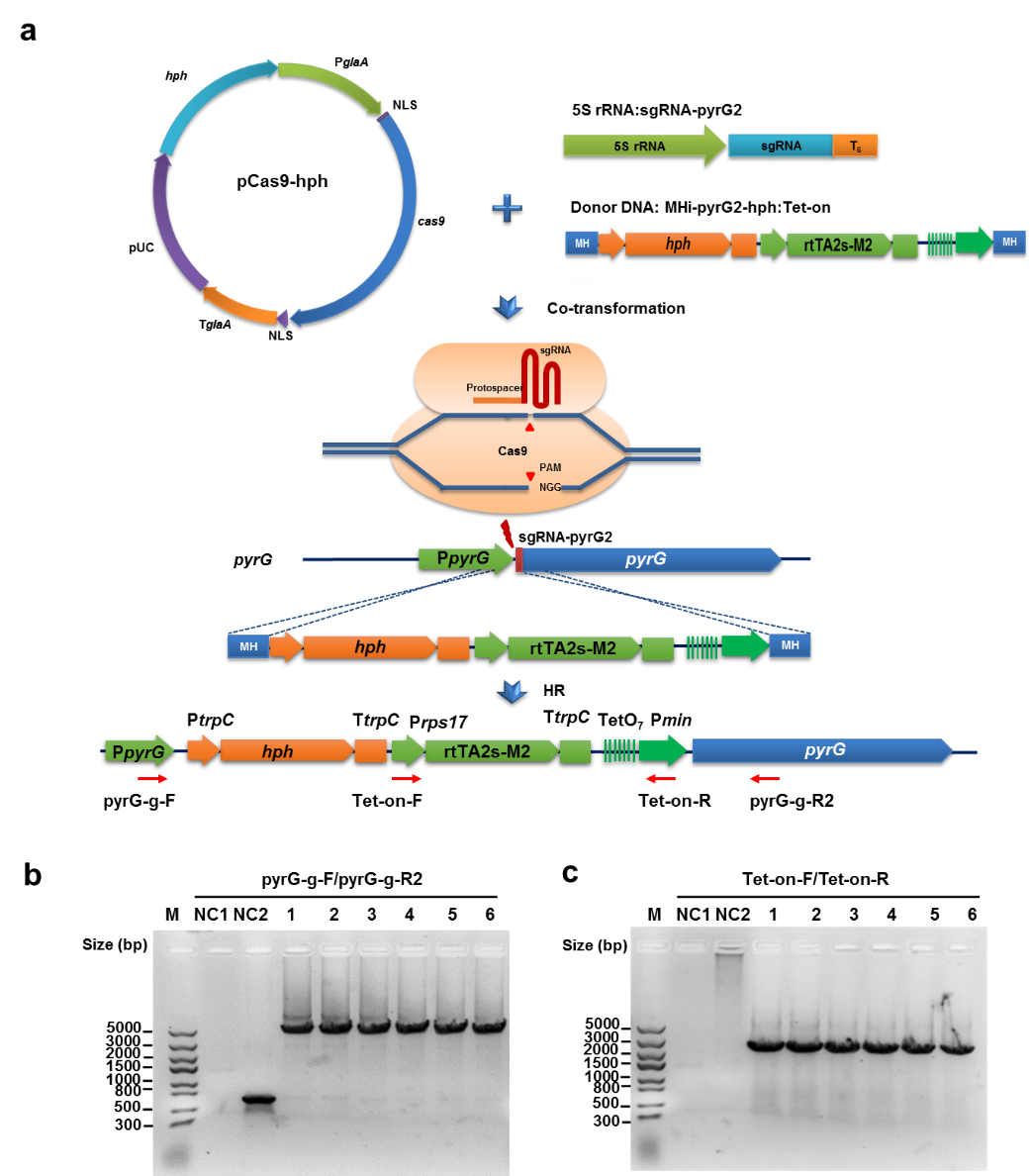


Figure S3 Titratable expression of *pyrG* mutagenesis constructed in *A. niger*

(a) Schematic diagram of *pyrG* titratalbe expression mutagenesis mediated by integrating the donor DNA with 40-bp micro-homology arms via CRISPR/Cas9 system based 5S rRNA. The donor DNA MHi-pyrG2-hyh:Tet-on, containing the Tet-on cassette, were co-transformed with linear sgRNA construct sgRNA-pyrG2 and Cas9 expression plasmid pCas9-hyh into the protoplasts of *A. niger* D353. DSBs at the locus of the upstream of *pyrG* encoding sequences, were generated by the Cas9 under the guide of sgRNA-pyrG2, and then were repaired by HR with the integration of donor DNA MHi-pyrG2-hyh:Tet-on, resulting in the replacement of *pyrG* native promoter. (b-c) Diagnostic PCR analysis of the *pyrG* titratable expression transformants. The expected sizes of PCR products of the mutants were 5424-bp (pyrG-g-F/pyrG-g-R2), when donor DNA MHi-pyrG2-hyh:Tet-on were correctly inserted at the expected loci of *pyrG*. The expected sizes of PCR products of the hosts were 1004-bp (pyrG-g-F/pyrG-g-R2), when the donor DNA was not inserted.

**
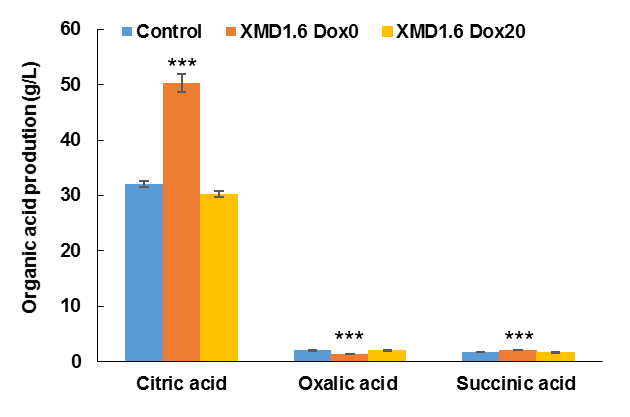
**

Figure S4 Extracellular organic acids of *pyrG* titratable expression mutant in 5 L bioreactor

Extracellular organic acids of *pyrG* titratable expression mutant under 0 and 20μg/ml Dox were compared with its parent strains in the 5 L bioreactor. 1 × 10^5^ spores/ml were inoculated in 3 L citrate fermentation (CitFM) media at 34°C in the dark for 124 hours. The extracellular organic acids were determined by the method of HPLC. Control, the parent strains D353; XMD1.6, the *pyrG* conditional expression mutants. Pairwise Student‘s t-tests were conducted between conditional expression mutant relative to the parent strains. *p* values are indicated as (<0.05, *; <0.01, **; <0.001, ***).

## Table S1 Protospacers used in this study

| Name | Protospacer Sequence (5' to 3') ^a^ | PAM |
| --- | --- | --- |
| sgRNA-albA | AGTGGGATCTCAAGAACTAC | TGG |
| sgRNA-kusA | CGAGCACTGGTAGATGATGA | AGG |
| sgRNA-pyrG1 | GAAGACCAATGTGACTGTCT | CGG |
| sgRNA-pyrG2 | GAGTAGTTCGAAGTTTCGAC | TGG |

Note: ^a^, sgRNA target sequences were predicted by a software package sgRNAcas9^1^ (<https://sourceforge.net/projects/sgrnacas9/>). sgRNAcas9 enables the fast design of sgRNA target sequences with minimized off-target effects by predicting the genome-wide Cas9 potential off-target cleavage sites (POT). The criteria for selection of efficient sgRNAs were instituted: (1) G/C content between 40% and 60%; (2) no more than 1 POT sites; (3) no more than 4 continuous T nucleotides (4,6 nucleotide poly (T) tract acts as a termination signal for RNA pol III), or other homopolymer sequences (more than 5 continuous A or C or G, more than 6 dinucleotide or trinucleotide repeats); (4) no *Bbs*I restriction site.

## Table S2 Primers used in this study

| Primer name | Primer Sequence (5' to 3') |
| --- | --- |
| \| Primers to construct Cas9 expressing plasmid pCas9-hyh \| \| --- \| | |
| PtrpC-Fm | GACGTTAACTGATATTGAAGGAGC |
| TtrpC-Rm | AACCCAGGGGCTGGTGACGG |
| pGm-Rrev | gctccttcaatatcagttaacgtcGGGTACCGAGCTCGAATTCGTAATCATG |
| pGm-Frev | ccgtcaccagcccctgggttGGTACCCTAGAGTCGAGGATTGCCTGAAC |
| \| Primers to construct pSM-AnpyrG \| \| --- \| | |
| AnpyrG-F | ATGTCTTCGAAGTCCCACCTCC |
| AnpyrG-R | TCAAAGTCCAACTCTTTTCTCG |
| TtrpC-Frev | cgagaaaagagttggactttgaAGTAGATGCCGACCGGATC |
| PtrpC-Rrev | gaggtgggacttcgaagacatATCGATGCTTGGGTAGAATAG |
| \| Primers to construct targeting sgRNA plasmids \| \| --- \| | |
| sgRNA-kusA-F | caccCGAGCACTGGTAGATGATGA |
| sgRNA-kusA-R | aaacTCATCATCTACCAGTGCTCG |
| sgRNA-pyrG1-F | caccGAAGACCAATGTGACTGTCT |
| sgRNA-pyrG1-R | aaacAGACAGTCACATTGGTCTTC |
| sgRNA-pyrG2-F | caccGAGTAGTTCGAAGTTTCGAC |
| sgRNA-pyrG2-R | aaacGTCGAAACTTCGAACTACTC |
| \| Primers to construct donor DNAs with micro-homologue arms \| \| --- \| | |
| MHi-sgRNA-albA-Fm | cctccgcctcccagcctacaagtgggatctcaagaactacGACGTTAACTGATATTGAAGGAGC |
| MHi-sgRNA-albA-Rm | gagcgcccttgctcaggcagaagttgttggtatagggaatAACCCAGGGGCTGGTGACGG |
| MHi-sgRNA-kusA-Fm | ctcatgaggtcaaaggacttcgagcactggtagatgatgaGACGTTAACTGATATTGAAG |
| MHi-sgRNA-kusA-Rm | gacctgctctttcgctggagatagaacctcccttgagtctAACCCAGGGGCTGGTGACGG |
| MHi-sgRNA-pyrG1-Fm | ttcgagattgccgaggccaagaagaccaatgtgactgtctGACGTTAACTGATATTGAAGGAGC |
| MHi-sgRNA-pyrG1-Rm | tcagcaagatctagtagctccttagtggtggtaacgtcagAACCCAGGGGCTGGTGACGG |
| MHi-sgRNA-pyrG2-Fm | gtatccgcgcacgtctctggatttacgaatcagggtccaGACGTTAACTGATATTGAAG |
| MHi-sgRNA-pyrG2-Rm | ggcacgggcagtgtaggtcaatcgcgacttggaggacatGGTGTTTAAACGGTGATGTC |
| Primers for diagnostic PCR of transformants | |
| kusA-g-F | AGACTGTACGCAAATTTACCCA |
| kusA-g-R | GTCGCCGCTTTTGAATCAGA |
| pyrG-g-F | CATGTGCAGCAGGGAATACGAG |
| pyrG-g-R1 | GTTTCCGCTTCCGTATCCGTTG |
| pyrG-g-R2 | GAGCCTTAAGTCCCTCAATGGTC |

Notes: Restriction sites are underlined. Fm represents forward primer with modification and Rm represents reverse primer with modification. The modified additional sequences were represented in lowercase letters.

## Table S3 DNA sequences of sgRNA constructs used in this study

| sgRNA constructs | DNA Sequence (5’ to 3’) |
| --- | --- |
| sgRNA6.1 (5S rRNA:sgRNA-albA) | CACATACGACCACAGGGTGTGGAAAACAGGGCTTCCCGTCCGCTCAGCCGTACTTAAGCCACACGCCGGGAGGTTAGTAGTTGGGTGGGTGACCACCAGCGAATCCCTTCTGTTGTATGAAAGGACGAAACACCAGTGGGATCTCAAGAACTACGTTTTAGAGCTAGAAATAGCAAGTTAAAATAAGGCTAGTCCGTTATCAACTTGAAAAAGTGGCACCGAGTCGGTGCTTTTTT |
| sgRNA6.13 (5S rRNA:sgRNA-kusA) | CACATACGACCACAGGGTGTGGAAAACAGGGCTTCCCGTCCGCTCAGCCGTACTTAAGCCACACGCCGGGAGGTTAGTAGTTGGGTGGGTGACCACCAGCGAATCCCTTCTGTTGTATGAAAGGACGAAACACCCGAGCACTGGTAGATGATGAGTTTTAGAGCTAGAAATAGCAAGTTAAAATAAGGCTAGTCCGTTATCAACTTGAAAAAGTGGCACCGAGTCGGTGCTTTTTT |
| sgRNA6.14 (5S rRNA:sgRNA-pyrG1) | CACATACGACCACAGGGTGTGGAAAACAGGGCTTCCCGTCCGCTCAGCCGTACTTAAGCCACACGCCGGGAGGTTAGTAGTTGGGTGGGTGACCACCAGCGAATCCCTTCTGTTGTATGAAAGGACGAAACACC GAAGACCAATGTGACTGTCTGTTTTAGAGCTAGAAATAGCAAGTTAAAATAAGGCTAGTCCGTTATCAACTTGAAAAAGTGGCACCGAGTCGGTGCTTTTTT |
| sgRNA6.15 (5S rRNA:sgRNA-pyrG2) | CACATACGACCACAGGGTGTGGAAAACAGGGCTTCCCGTCCGCTCAGCCGTACTTAAGCCACACGCCGGGAGGTTAGTAGTTGGGTGGGTGACCACCAGCGAATCCCTTCTGTTGTATGAAAGGACGAAACACC GAGTAGTTCGAAGTTTCGACGTTTTAGAGCTAGAAATAGCAAGTTAAAATAAGGCTAGTCCGTTATCAACTTGAAAAAGTGGCACCGAGTCGGTGCTTTTTT |

Note: Green letters indicate the promoter region for sgRNA expression. Blue letters indicate the sgRNA scaffold. Red letters indicate the terminator of *RNU6* gene. Red underlined letters indicate genetic targets.

## Table S4 DNA sequences of donor DNAs used in this study

| Donor DNA | | DNA Sequence (5’ to 3’) | |
| --- | --- | --- | --- |
| MHi-albA-AnpyrG | | CCTCCGCCTCCCAGCCTACAAGTGGGATCTCAAGAACTACgacgttaactgatattgaaggagcactttttgggcttggctggagctagtggaggtcaacaatgaatgcctattttggtttagtcgtccaggcggtgagcacaaaatttgtgtcgtttgacaagatggttcatttaggcaactggtcagatcagccccacttgtagcagtagcggcggcgctcgaagtgtgactcttattagcagacaggaacgaggacattattatcatctgctgcttggtgcacgataacttggtgcgtttgtcaagcaaggtaagtgaacgacccggtcataccttcttaagttcgcccttcctccctttatttcagattcaatctgacttacctattctacccaagcatcgatATGTCTTCGAAGTCCCACCTCCCCTACGCAATTCGCGCAACCAACCATCCCAACCCTTTAACATCTAAACTCTTCTCCATCGCCGAGGAGAAGAAAACCAACGTCACCGTCTCCGCAGACGTTACTACTTCCGCCGAGCTCCTCGATCTTGCTGACCGTACATCCTGCACCAATGCCCCTCCAGGATAACAAATAGCTGATGCGTAGTGAGTACAGGCCTAGGCCCCTATATCGCAGTTCTGAAAACCCACATCGACATCCTCACCGATCTCACCCCGTCGACCCTTTCCTCGCTCCAATCCCTCGCGACAAAGCACAACTTCCTCATCTTTGAGGACCGCAAGTTCATCGACATCGGCAACACCGTGCAAAAGCAGTACCACGGTGGCGCTCTCCGCATCTCCGAATGGGCACACATCATCAACTGCGCCATCCTGCCGGGCGAAGGGATCGTCGAGGCCCTCGCACAGACAACCAAGTCTCCTGACTTTAAAGACGCGAATCAACGAGGTCTCCTGATTCTTGCCGAGATGACGAGTAAGGGATCTCTTGCGACAGGGGAGTACACGGCACGCTCGGTTGAGTACGCGCGGAAGTATAAGGGGTTTGTGATGGGATTCGTGAGTACAAGGGCGTTGAGTGAGGTGCTGCCCGAACAGAAAGAGGAGAGCGAGGATTTTGTCGTCTTTACGACTGGGGTGAATCTGTCGGATAAGGGGGATAAGCTGGGGCAGCAGTATCAGACACCTGGGTCGGCGGTTGGGCGAGGTGCGGACTTTATCATTGCGGGTAGGGGCATCTATAAGGCGGACGATCCAGTCGAGGCGGTTCAGAGGTACCGGGAGGAAGGCTGGAAAGCTTACGAGAAAAGAGTTGGACTTTGAagtagatgccgaccggatcgggcccgatccacttaacgttactgaaatcatcaaacagcttgacgaatctggatataagatcgttggtgtcgatgtcagctccggagttgagacaaatggtgttcaggatctcgataagatacgttcatttgtccaagcagcaaagagtgccttctagtgatttaatagctccatgtcaacaagaataaaacgcgtttcgggtttacctcttccagatacagctcatctgcaatgcattaatgcattggacctcgcaaccctagtacgcccttcaggctccggcgaagcagaagaatagcttagcagagtctattttcattttcgggagacgagatcaagcagatcaacggtcgtcaagagacctacgagactgaggaatccgctcttggctccacgcgactatatatttgtctctaattgtactttgacatgctcctcttctttactctgatagcttgactatgaaaattccgtcaccagcccctgggttATTCCCTATACCAACAACTTCTGCCTGAGCAAGGGCGCTC | |
| MHi-kusA-hph | | CTCATGAGGTCAAAGGACTTCGAGCACTGGTAGATGATGAgacgttaactgatattgaaggagcactttttgggcttggctggagctagtggaggtcaacaatgaatgcctattttggtttagtcgtccaggcggtgagcacaaaatttgtgtcgtttgacaagatggttcatttaggcaactggtcagatcagccccacttgtagcagtagcggcggcgctcgaagtgtgactcttattagcagacaggaacgaggacattattatcatctgctgcttggtgcacgataacttggtgcgtttgtcaagcaaggtaagtgaacgacccggtcataccttcttaagttcgcccttcctccctttatttcagattcaatctgacttacctattctacccaagcatcgatATGAAAAAGCCTGAACTCACCGCGACGTCTGTCGAGAAGTTTCTGATCGAAAAGTTCGACAGCGTCTCCGACCTGATGCAGCTCTCGGAGGGCGAAGAATCTCGTGCTTTCAGCTTCGATGTAGGAGGGCGTGGATATGTCCTGCGGGTAAATAGCTGCGCCGATGGTTTCTACAAAGATCGTTATGTTTATCGGCACTTTGCATCGGCCGCGCTCCCGATTCCGGAAGTGCTTGACATTGGGGAATTCAGCGAGAGCCTGACCTATTGCATCTCCCGCCGTGCACAGGGTGTCACGTTGCAAGACCTGCCTGAAACCGAACTGCCCGCTGTTCTGCAGCCGGTCGCGGAGGCCATGGATGCGATCGCTGCGGCCGATCTTAGCCAGACGAGCGGGTTCGGCCCATTCGGACCGCAAGGAATCGGTCAATACACTACATGGCGTGATTTCATATGCGCGATTGCTGATCCCCATGTGTATCACTGGCAAACTGTGATGGACGACACCGTCAGTGCGTCCGTCGCGCAGGCTCTCGATGAGCTGATGCTTTGGGCCGAGGACTGCCCCGAAGTCCGGCACCTCGTGCACGCGGATTTCGGCTCCAACAATGTCCTGACGGACAATGGCCGCATAACAGCGGTCATTGACTGGAGCGAGGCGATGTTCGGGGATTCCCAATACGAGGTCGCCAACATCTTCTTCTGGAGGCCGTGGTTGGCTTGTATGGAGCAGCAGACGCGCTACTTCGAGCGGAGGCATCCGGAGCTTGCAGGATCGCCGCGGCTCCGGGCGTATATGCTCCGCATTGGTCTTGACCAACTCTATCAGAGCTTGGTTGACGGCAATTTCGATGATGCAGCTTGGGCGCAGGGTCGATGCGACGCAATCGTCCGATCCGGAGCCGGGACTGTCGGGCGTACACAAATCGCCCGCAGAAGCGCGGCCGTCTGGACCGATGGCTGTGTAGAAGTACTCGCCGATAGTGGAAACCGACGCCCCAGCACTCGTCCGAGGGCAAAGGAATAGagtagatgccgaccggatcgatccacttaacgttactgaaatcatcaaacagcttgacgaatctggatataagatcgttggtgtcgatgtcagctccggagttgagacaaatggtgttcaggatctcgataagatacgttcatttgtccaagcagcaaagagtgccttctagtgatttaatagctccatgtcaacaagaataaaacgcgtttcgggtttacctcttccagatacagctcatctgcaatgcattaatgcattggacctcgcaaccctagtacgcccttcaggctccggcgaagcagaagaatagcttagcagagtctattttcattttcgggagacgagatcaagcagatcaacggtcgtcaagagacctacgagactgaggaatccgctcttggctccacgcgactatatatttgtctctaattgtactttgacatgctcctcttctttactctgatagcttgactatgaaaattccgtcaccagcccctgggttAGACTCAAGGGAGGTTCTATCTCCAGCGAAAGAGCAGGTC | |
| MHi-pyrG1-hph | | TTCGAGATTGCCGAGGCCAAGAAGACCAATGTGACTGTCTgacgttaactgatattgaaggagcactttttgggcttggctggagctagtggaggtcaacaatgaatgcctattttggtttagtcgtccaggcggtgagcacaaaatttgtgtcgtttgacaagatggttcatttaggcaactggtcagatcagccccacttgtagcagtagcggcggcgctcgaagtgtgactcttattagcagacaggaacgaggacattattatcatctgctgcttggtgcacgataacttggtgcgtttgtcaagcaaggtaagtgaacgacccggtcataccttcttaagttcgcccttcctccctttatttcagattcaatctgacttacctattctacccaagcatcgatATGAAAAAGCCTGAACTCACCGCGACGTCTGTCGAGAAGTTTCTGATCGAAAAGTTCGACAGCGTCTCCGACCTGATGCAGCTCTCGGAGGGCGAAGAATCTCGTGCTTTCAGCTTCGATGTAGGAGGGCGTGGATATGTCCTGCGGGTAAATAGCTGCGCCGATGGTTTCTACAAAGATCGTTATGTTTATCGGCACTTTGCATCGGCCGCGCTCCCGATTCCGGAAGTGCTTGACATTGGGGAATTCAGCGAGAGCCTGACCTATTGCATCTCCCGCCGTGCACAGGGTGTCACGTTGCAAGACCTGCCTGAAACCGAACTGCCCGCTGTTCTGCAGCCGGTCGCGGAGGCCATGGATGCGATCGCTGCGGCCGATCTTAGCCAGACGAGCGGGTTCGGCCCATTCGGACCGCAAGGAATCGGTCAATACACTACATGGCGTGATTTCATATGCGCGATTGCTGATCCCCATGTGTATCACTGGCAAACTGTGATGGACGACACCGTCAGTGCGTCCGTCGCGCAGGCTCTCGATGAGCTGATGCTTTGGGCCGAGGACTGCCCCGAAGTCCGGCACCTCGTGCACGCGGATTTCGGCTCCAACAATGTCCTGACGGACAATGGCCGCATAACAGCGGTCATTGACTGGAGCGAGGCGATGTTCGGGGATTCCCAATACGAGGTCGCCAACATCTTCTTCTGGAGGCCGTGGTTGGCTTGTATGGAGCAGCAGACGCGCTACTTCGAGCGGAGGCATCCGGAGCTTGCAGGATCGCCGCGGCTCCGGGCGTATATGCTCCGCATTGGTCTTGACCAACTCTATCAGAGCTTGGTTGACGGCAATTTCGATGATGCAGCTTGGGCGCAGGGTCGATGCGACGCAATCGTCCGATCCGGAGCCGGGACTGTCGGGCGTACACAAATCGCCCGCAGAAGCGCGGCCGTCTGGACCGATGGCTGTGTAGAAGTACTCGCCGATAGTGGAAACCGACGCCCCAGCACTCGTCCGAGGGCAAAGGAATAGagtagatgccgaccggatcgatccacttaacgttactgaaatcatcaaacagcttgacgaatctggatataagatcgttggtgtcgatgtcagctccggagttgagacaaatggtgttcaggatctcgataagatacgttcatttgtccaagcagcaaagagtgccttctagtgatttaatagctccatgtcaacaagaataaaacgcgtttcgggtttacctcttccagatacagctcatctgcaatgcattaatgcattggacctcgcaaccctagtacgcccttcaggctccggcgaagcagaagaatagcttagcagagtctattttcattttcgggagacgagatcaagcagatcaacggtcgtcaagagacctacgagactgaggaatccgctcttggctccacgcgactatatatttgtctctaattgtactttgacatgctcctcttctttactctgatagcttgactatgaaaattccgtcaccagcccctgggttCTGACGTTACCACCACTAAGGAGCTACTAGATCTTGCTGA | |
| MHi-pyrG2-hyh:Tet-on | GTATCCGCGCACGTCTCTGGATTTACGAATCAGGGTCCAgacgttaactgatattgaaggagcactttttgggcttggctggagctagtggaggtcaacaatgaatgcctattttggtttagtcgtccaggcggtgagcacaaaatttgtgtcgtttgacaagatggttcatttaggcaactggtcagatcagccccacttgtagcagtagcggcggcgctcgaagtgtgactcttattagcagacaggaacgaggacattattatcatctgctgcttggtgcacgataacttggtgcgtttgtcaagcaaggtaagtgaacgacccggtcataccttcttaagttcgcccttcctccctttatttcagattcaatctgacttacctattctacccaagcatcgatATGAAAAAGCCTGAACTCACCGCGACGTCTGTCGAGAAGTTTCTGATCGAAAAGTTCGACAGCGTCTCCGACCTGATGCAGCTCTCGGAGGGCGAAGAATCTCGTGCTTTCAGCTTCGATGTAGGAGGGCGTGGATATGTCCTGCGGGTAAATAGCTGCGCCGATGGTTTCTACAAAGATCGTTATGTTTATCGGCACTTTGCATCGGCCGCGCTCCCGATTCCGGAAGTGCTTGACATTGGGGAATTCAGCGAGAGCCTGACCTATTGCATCTCCCGCCGTGCACAGGGTGTCACGTTGCAAGACCTGCCTGAAACCGAACTGCCCGCTGTTCTGCAGCCGGTCGCGGAGGCCATGGATGCGATCGCTGCGGCCGATCTTAGCCAGACGAGCGGGTTCGGCCCATTCGGACCGCAAGGAATCGGTCAATACACTACATGGCGTGATTTCATATGCGCGATTGCTGATCCCCATGTGTATCACTGGCAAACTGTGATGGACGACACCGTCAGTGCGTCCGTCGCGCAGGCTCTCGATGAGCTGATGCTTTGGGCCGAGGACTGCCCCGAAGTCCGGCACCTCGTGCACGCGGATTTCGGCTCCAACAATGTCCTGACGGACAATGGCCGCATAACAGCGGTCATTGACTGGAGCGAGGCGATGTTCGGGGATTCCCAATACGAGGTCGCCAACATCTTCTTCTGGAGGCCGTGGTTGGCTTGTATGGAGCAGCAGACGCGCTACTTCGAGCGGAGGCATCCGGAGCTTGCAGGATCGCCGCGGCTCCGGGCGTATATGCTCCGCATTGGTCTTGACCAACTCTATCAGAGCTTGGTTGACGGCAATTTCGATGATGCAGCTTGGGCGCAGGGTCGATGCGACGCAATCGTCCGATCCGGAGCCGGGACTGTCGGGCGTACACAAATCGCCCGCAGAAGCGCGGCCGTCTGGACCGATGGCTGTGTAGAAGTACTCGCCGATAGTGGAAACCGACGCCCCAGCACTCGTCCGAGGGCAAAGGAATAGagtagatgccgaccggatcgatccacttaacgttactgaaatcatcaaacagcttgacgaatctggatataagatcgttggtgtcgatgtcagctccggagttgagacaaatggtgttcaggatctcgataagatacgttcatttgtccaagcagcaaagagtgccttctagtgatttaatagctccatgtcaacaagaataaaacgcgtttcgggtttacctcttccagatacagctcatctgcaatgcattaatgcattggacctcgcaaccctagtacgcccttcaggctccggcgaagcagaagaatagcttagcagagtctattttcattttcgggagacgagatcaagcagatcaacggtcgtcaagagacctacgagactgaggaatccgctcttggctccacgcgactatatatttgtctctaattgtactttgacatgctcctcttctttactctgatagcttgactatgaaaattccgtcaccagcccctgggttgcggccgctctagaaccctcggctggtctgtcttacacaagatcacacgctttgatctacaatcaccccaagtatggacctcaggcccaccaacgcccagtggaggcacgtatcttgcgccccagaggacggaagggcaagaacacgaaggccattgcaggtgtggctggtattgccgtggaagacttgaatactgtcacctttaccgagcaggactctccggccggccttgcctactttgacgcctctatccccggtggtgctaagtactgggccacccctatccgggcctttgtcgactcggaaggcaagattggcctggcttcttaccgcgctagcgctactgctaaagccccttacggaatcgatagccagaagaagcccggatcgtacagcatctctgacgtggctcgcggcgaccagcgcgtggtcccgagactggatagacagcggaacgccgcagcggagactgaggaggttgcgcgtaacctcatgaagagccttggctcctagaatacaggtgtttcagtcccaagcaatttttgtctcatgtgtcagacatgttctatcttccacttagagagctttgctgtatgattaggtttttggatggtaaatgtatagttattttcgcacttgttctcatttgtttgtgatataagccggctacatctcattctaagtataaattaaattgcaatttcaaagacgtctcagtaattactgatcactataaacctggttttgccataccgtaaggcaacagccatcttcataactaactagtccgttacatatgaaatacataaatggcgcttaataatccagcgcaacgcccgaccgctttgacaggtaagcgcacgtgacattaaatagggcatccacatcagggtagatacgcatcacatgacccgcacatcctaagcgagggacgggtaagttgaaggagcgaaagacatcagaacatcttggacggtttttcgaacatccatccagccagcaacaaaccgccaaaGAATTCACCATGTCTAGACTGGACAAGAGCAAAGTCATAAACGGCGCTCTGGAATTACTCAATGGAGTCGGTATCGAAGGCCTGACGACAAGGAAACTCGCTCAAAAGCTGGGAGTTGAGCAGCCTACCCTGTACTGGCACGTGAAGAACAAGCGGGCCCTGCTCGATGCCCTGCCAATCGAGATGCTGGACAGGCATCATACCCACTTCTGCCCCCTGGAAGGCGAGTCATGGCAAGACTTTCTGCGGAACAACGCCAAGTCATTCCGCTGTGCTCTCCTCTCACATCGCGACGGGGCTAAAGTGCATCTCGGCACCCGCCCAACAGAGAAACAGTACGAAACCCTGGAAAATCAGCTCGCGTTCCTGTGTCAGCAAGGCTTCTCCCTGGAGAACGCACTGTACGCTCTGTCCGCCGTGGGCCACTTTACACTGGGCTGCGTATTGGAGGAACAGGAGCATCAAGTAGCAAAAGAGGAAAGAGAGACACCTACCACCGATTCTATGCCCCCACTTCTGAGACAAGCAATTGAGCTGTTCGACCGGCAGGGAGCCGAACCTGCCTTCCTTTTCGGCCTGGAACTAATCATATGTGGCCTGGAGAAACAGCTAAAGTGCGAAAGCGGCGGGCCGGCCGACGCCCTTGACGATTTTGACTTAGACATGCTCCCAGCCGATGCCCTTGACGACTTTGACCTTGATATGCTGCCTGCTGACGCTCTTGACGATTTTGACCTTGACATGCTCCCCgggtaactaagtaaggatccactagtacagcagaagaatctctctccgctgttgcttcagtgtctgccatgcattaacttcatcctactgtcctacccgcagtacccattcacatttgcggcagataccaggttgttttctattccctcggttcttcagttcttcagatattacattactcgagaaattggcggcgatgcaggagtttggttggttttaattgtttcagtctccttgacctgatttgtatgacatgcaatgtttcgacggaagactatctcggtgaatatacgtcatgaatcatgcggccgcgcgtatcacgaggccctttcgtcttcactcgagtttaccactccctatcagtgatagagaaaagtgaaagtcgagtttaccactccctatcagtgatagagaaaagtgaaagtcgagtttaccactccctatcagtgatagagaaaagtgaaagtcgagtttaccactccctatcagtgatagagaaaagtgaaagtcgagtttaccactccctatcagtgatagagaaaagtgaaagtcgagtttaccactccctatcagtgatagagaaaagtgaaagtcgagtttaccactccctatcagtgatagagaaaagtgaaagtcgagctccccatcttcagtatattcatcttcccatcc*aagaacctttatttcccctaagtaagtactttgctacatccatactccatccttcccatcccttattcctttgaacctttcagttcgagctttcccacttcatcgcagcttgactaacagctaccccgcttgagcagacatcaccgtttaaac*accATGTCCTCCAAGTCGCGATTGACCTACACTGCCCGTGCC | |  |

Note: Blue letters indicate the homogenous arms located at the 5’ and 3’ flanking region of the genetic target sites. Black uppercase letters indicate the encoding sequences of selection marker *hph*. Green lowercase letters indicate the promoter P*trpC* in selection marker *hph* expression cassette. Orange lowercase letters indicate the terminator T*trpC* in selection marker expression *hph* cassette. Black underlined uppercase letters indicate the encoding sequences of the regulator rtRA2s-M2 in Tet-on system. Green underlined lowercase letters indicate the promoter P*fraA* in rtRA2s-M2 expression cassette. Orange underlined lowercase letters indicate the terminator T*cgrA* in rtRA2s-M2 expression cassette. Purple lowercase letters indicate the rtRA2s-M2 binding site Tet0_7_. Green italic lowercase letters indicate the promoter P*min* for the targeted gene.
